# Is altitude‐induced polycythaemia an unintended evolutionary mistake?

**DOI:** 10.1113/EP093735

**Published:** 2026-03-15

**Authors:** Peter D. Wagner, Tatum S. Simonson

**Affiliations:** ^1^ Department of Medicine, Division of Pulmonary, Critical Care, Sleep Medicine, and Physiology University of California San Diego La Jolla California USA

Have you ever wondered why, when sea‐level folk travel to altitude, haemoglobin concentration ([Hb]) rises? We have, and the more we ponder it, the more it seems to be an evolutionary mistake – the opposite of what we have been taught. Standard teaching is that with less O_2_ in the air at altitude, [Hb] increases to compensate, partially (perhaps even fully) restoring arterial O_2_ concentration and thus potentially also O_2_ transport. Moreover, at sea level, ${\dot{V}}_{O_{2}\max}$ is lower in the same person when [Hb] is acutely reduced (keeping blood volume constant) (Schaffartzik et al., 1993). This raises the possibility that ${\dot{V}}_{O_{2}\max}$ will increase with time at altitude as [Hb] is elevated.

That polycythaemia gedevelops at altitude is undisputed, but the questions are (a) whether any selective pressure existed to preserve this response, and if so, what that pressure was, and (b) whether it also restores O_2_‐dependent bodily functions, in particular reproductive success and exercise capacity. This article will make the case that altitude polycythaemia is unhelpful to both and is therefore an evolutionary biological error. We will conclude with speculation on how this came to be. We alert the reader that this article is not about the merits of altitude training for competitive athletes. It should not be used to argue for or against altitude training, which adds factors such as blood volume and Hb mass, hyperventilation, acid–base regulation and hydration control, training load and adaptive gene expression. Any of these may differ compared to sea level and thus also affect exercise performance.

Consider this first: a fundamental premise of evolution is the conservation of random mutations whose physiological consequences confer survival benefit, along with the loss of mutations that do not: natural selection. But for a random mutation to result in a benefit and spread through the population, there must have been anti‐survival pressure in the first place, such that absent that mutation, survival would be less: selective pressure.

How does this translate to polycythaemia at altitude? Almost 95% of the world's population resides below 1524 m (5000 ft) elevation, that is, *in the absence of significant hypoxic anti‐survival selective pressure*. How could we have developed a polycythaemic response to a hypoxic environment we have never inhabited? Yet we do have that response – most low‐altitude residents will develop polycythaemia when ascending to altitude. It is a very strongly conserved response. But a response of what origin and to what end?

This conundrum came into focus for one of us (P.D.W.) in 1998 during Bengt Saltin's research expedition to Mt Chacaltaya in Bolivia (Figure 1a, b). At 5260 m, the altitude is roughly that of the Everest Base Camp (5364 m), but at Chacaltaya there used to be a fancy ski resort. Sadly, the glacier melted and wiped out the sport there, leaving a large empty structure that could be filled with researchers and study participants in more comfort and allowing more research complexity than Base Camp. The road in is not too bad, and well‐packed scientific equipment arrives in good shape. There are even electricity and plumbing.

**FIGURE 1:**
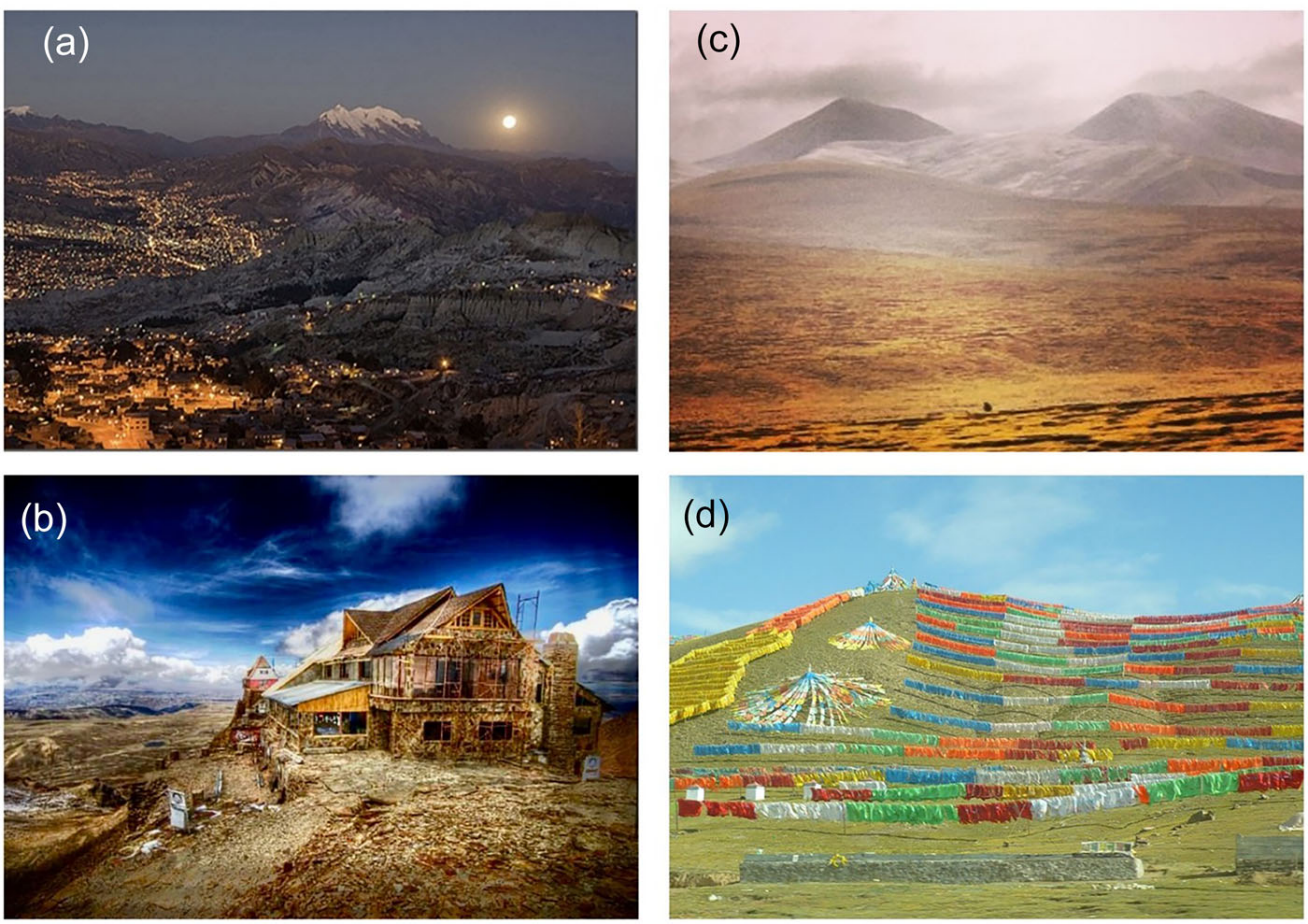
(a, b) Mount Chacaltaya and research station at 5260 m in Bolivian Andes. (c, d) Maduo county at 4200–4300 m in the Tibetan Plateau.

Because he knew he would likely not tolerate this altitude for more than a few days, Bengt had asked P.D.W. to be a part of the expedition as a proxy to carry on should he have to descend. Unfortunately, Bengt was correct. Big shoes to fill. It helped that P.D.W. had an ulterior motive: understanding the consequences of his perennially low‐side [Hb] for exercise at altitude. Planning a 6‐h cycling trip with his wife from toes‐in‐the‐ocean to 36 miles up Haleakala (3055 m; 10,023 ft on Maui), he wanted to know if he could do it with a low [Hb]. In the end, he (at age 72) and Harrieth (age undisclosed) made it up without incident, his [Hb] still low. E‐bikes were not yet a thing, in case you were wondering if they cheated.

Bengt's expedition was a 9‐week study of the effects of this 5260 m altitude on endurance exercise capacity in healthy, young, recreationally fit sea level residents. Two of the many experiments conducted addressed the issue of how altitude polycythaemia affected ${\dot{V}}_{O_{2}\max}$. One was comparison of ${\dot{V}}_{O_{2}\max}$ at the start and end of the 9 weeks, with both studies carried out at 5260 m (Calbet et al., 2002). The following numbers are for ${\dot{V}}_{O_{2}\max}$ at (a) sea level, (b) within a week of arrival and (c) 9 weeks still at 5260 m (Table 1). Clearly, a large increase in systemic O_2_ delivery over the 9 weeks enabled only a minimal increase in ${\dot{V}}_{O_{2}\max}$.

**TABLE 1:** Mean values for key oxygen transport variables at maximal exercise in sea level residents before and after ascent to Chacaltaya, Bolivia (5260 m)

	Sea level	5260 m arrival	5260 m after 9 weeks
Arterial [O_2_] (mL/dL)	19.1	13.2	19.2
Cardiac output (L/min)	23	19	20
Systemic O_2_ delivery (L/min)	4.4	2.5	3.9
${\dot{V}}_{O_{2}\max}$ (L/min)	4.1	2.2	2.4

While oxygen delivery returned almost to sea level values by 9 weeks, ${\dot{V}}_{O_{2}\max}$ remained similar to that seen immediately after arrival at altitude (Calbet et al., 2002).

The second study was to measure ${\dot{V}}_{O_{2}\max}$ near the end of the 9‐week sojourn before and an hour after isovolumic reduction of [Hb] to sea level values (from 18.5 to 14.2 g/dL). This intervention did not alter ${\dot{V}}_{O_{2}\max}$ (Calbet et al., 2004). We saw this second, 1‐day study as controlling for confounding factors like muscle mass loss and activity level changes over the 9 weeks. We also found that breathing 100% O_2_ immediately restored exercise capacity, showing that reduced exercise capacity after 9 weeks at altitude was indeed due to lower muscle mitochondrial O_2_ availability, not lower oxidative capacity (Calbet et al., 2003). Taken together, these studies told us that the high [Hb] did not confer an O_2_ utilization benefit. In Darwinian terms, the high [Hb] did not increase the chances of escaping a hungry sabretooth tiger: it was not likely naturally selective.

Rarely does one make a truly original observation. Indeed, Bob Winslow states in his 1987 book with the legendary Carlos Monge (p. 182, Winslow & Monge Cassinelli, 1987) that after acute reduction in [Hb], no exercise impairment occurred in previously polycythaemic high‐altitude Andeans. Subject number was small, insufficient data could be obtained (${\dot{V}}_{O_{2}}$ was not measured, only ${\dot{V}}_{CO_{2}}$), and so the results can only be taken as suggestive of no exercise benefit from polycythaemia.

Then more than a decade later in 2010, after it had been discovered that many lifelong residents of the Tibetan Plateau (average elevation 4500 m) had unexpectedly normal sea level [Hb] (Adams & Strang, 1975; Beall & Goldstein, 1987; Beall & Reichsman, 1984; Beall et al., 1998), two simultaneous 2010 *Science* papers (Simonson et al., 2010; Yi et al., 2010) and another in *PNAS* (Beall et al., 2010) from different groups studying this population showed associations with the hypoxia inducible factor (HIF) gene pathway critical to sensing hypoxia and turning on erythropoietin (amongst many other pathways). These were changes that could explain the loss of altitude polycythaemia. What was going on here? *The selective pressure of hypoxia was favouring those Tibetans with lower, not higher, [Hb]*. There is evidence that these HIF‐related changes may have already been present in the ancestors of the high‐altitude Tibetans (Huerta‐Sánchez et al., 2014). But this does not alter the fact that today most Tibetans living at 4200–4300 m display near‐sea level [Hb]. That remains the key observation.

The first author of one of these two *Science* papers (T.S.S., coauthor of the present article) was still a graduate student at the University of Utah at the time, but nearing thesis defence. She was exploring places for postdoctoral work and got in touch with the Physiology Faculty at the University of California San Diego. Somehow the conversation turned to why Tibetans were losing their polycythaemic response to altitude, when convention still stated that more Hb is better for O_2_ transport at altitude. Bengt Saltin's Chacaltaya studies led to the suggestion we build a team to go to Tibet and compare exercise capacity in Tibetans who showed essentially sea‐level [Hb] with that of Han Chinese also living long term on the Tibetan Plateau, but whose ancestry was at sea level and [Hb]‐high. Still a grad student, T.S.S. set up and secured funding for that study in cooperation with her Chinese collaborators from the *Science* paper, and so we all flew to China. We stayed in a remote region with rudimentary facilities (Figure 1c–d), but there was electricity – much of the time – and the study was done. As expected, the Han participants living at 4200 m were mostly polycythaemic, while most of the Tibetans had [Hb] values similar to those we see at sea level. The main finding was that ${\dot{V}}_{O_{2}\max}$ was higher in the Tibetans (lower [Hb]) compared to the Han Chinese (higher [Hb]) (Simonson et al., 2015). There was actually a decent, inverse, relationship between exercise capacity and [Hb] (Figure 2) when the data from both groups were combined.

**FIGURE 2:**
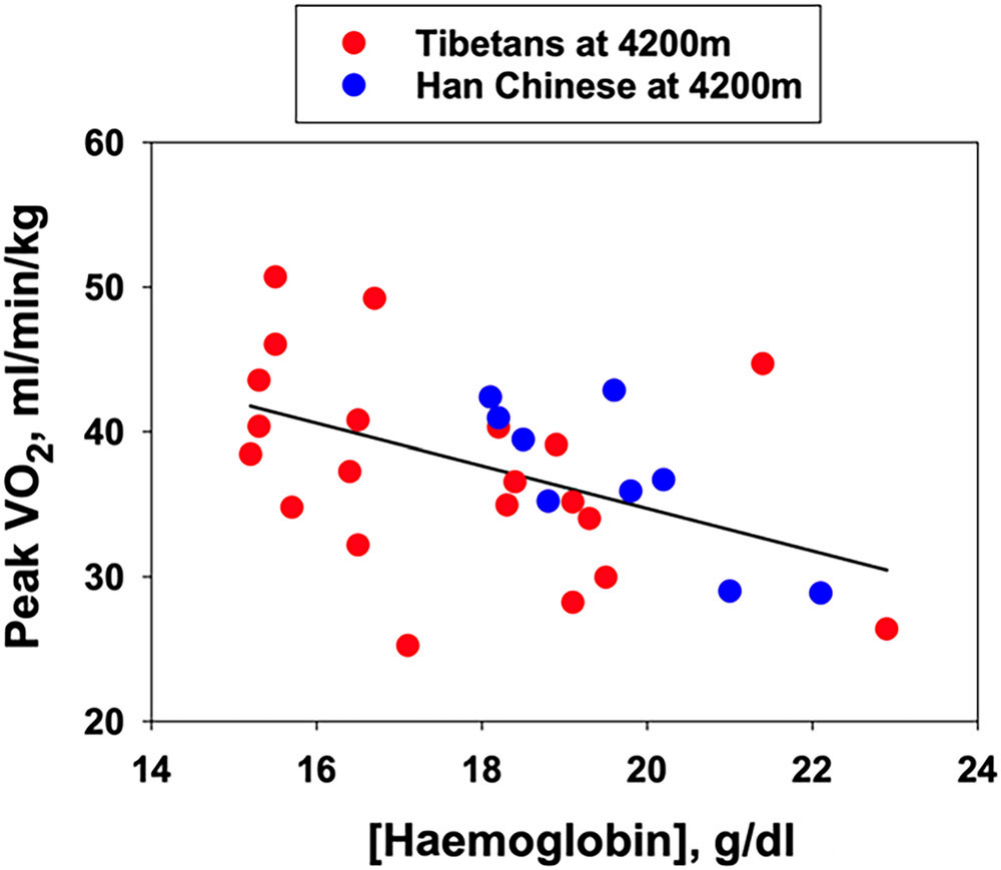
Inverse relationship between ${\dot{V}}_{O_{2}\max}$ and haemoglobin concentration in Tibetan men (red circles, *n* = 21) and Han Chinese men (blue circles, *n* = 9). The data further suggest a single relationship that is independent of Han Chinese or Tibetan background (modified from Simonson et al., 2015).

Subsequent studies in Andean men at 4340 m, including participants with and without excessive erythrocytosis, showed similar peak ${\dot{V}}_{O_{2}}$ both before and after isovolumic haemodilution (Anza‐Ramírez et al., 2023). Taken together, Schaffartzik's studies and those from Chacaltaya (both using individuals without high‐altitude ancestry) plus those from Tibet and from the Andes revealed the same lack of enhancement of ${\dot{V}}_{O_{2}\max}$ by high [Hb] in three different ancestral groups.

All experiments, including those at Chacaltaya and in Tibet, have limitations that can cloud interpretation of the outcomes, which is why most of us try and conduct research studies using various different approaches to answering the same question to see what findings may be common to all in spite of (different) limitations of any one approach. As these studies were accumulated, a clear story emerged that exercise capacity at altitude is not enhanced by polycythaemia.

However, this seems to clash with Schaffartzik's finding (Schaffartzik et al., 1993) that at sea level, ${\dot{V}}_{O_{2}\max}$ is raised by an increase in [Hb]. But Schaffartzik also exercised participants of his study in acute hypoxia and found that hypoxia essentially eliminated the sea level ${\dot{V}}_{O_{2}\max}$ reduction associated with the lower [Hb], which is consistent with our thesis.

To explain this, it should first be noted that Schaffartzik's 13.2% change in [Hb] led to a far smaller change in ${\dot{V}}_{O_{2}\max}$ – by 7.8% at sea level and just 0.8% in hypoxia. The smaller relative change in ${\dot{V}}_{O_{2}\max}$ than [Hb] at any altitude is due to opposing effects of [Hb] on the diffusive and convective components of O_2_ transport: a reduction in [Hb] will reduce convective O_2_ delivery to the muscle, but at the same time will allow greater fractional O_2_ extraction (for the same diffusing capacity). This higher O_2_ extraction partially offsets the reduced convective delivery (Piiper & Scheid, 1981; Wagner, 1996).

But why does the [Hb] effect on ${\dot{V}}_{O_{2}\max}$ essentially evaporate in hypoxia? The explanation is that while at sea level the O_2_ transport system is operating on both the flat and steep parts of the O_2_Hb dissociation curve, at altitude it is limited to the steep part of the curve and lower $P_{O_{2}}$ values. This in turn challenges diffusive steps in O_2_ transport more at altitude than at sea level because altitude results in smaller $P_{O_{2}}$ diffusion gradients in both lung and muscle. At altitude, this negative effect of a higher [Hb] on diffusion equilibration balances its beneficial effect on convective (perfusive) O_2_ transport resulting in no improvement in ${\dot{V}}_{O_{2}\max}$.

Returning to evolutionary considerations, the story was becoming somewhat confusing and counterintuitive: healthy sea‐level residents all have an altitude‐induced polycythaemic response, but without the selective pressure of hypoxia to generate it. Yet most Tibetan high‐altitude residents do not have this polycythaemic response despite strong selective pressure of hypoxia. This seemed crazy. Thus, one big question is why the majority of Tibetans living at altitude have little or no polycythaemic response to hypoxia. They certainly have the selective pressure of hypoxia living at 4200–4300 m. The differences in exercise capacity we found were significant, but not likely different enough to affect survival, given the absence of sabretooth tigers on the Tibetan Plateau. One place to look is in fetal and post‐natal development on the Tibetan Plateau. Lorna Moore and Cynthia Beall have separately documented higher reproductive success in Tibetans with lower [Hb] (Gonzales, 2009; Moore, 2001; Ye et al., 2024). Perhaps fetal oxygenation, neural development, and perinatal survival at altitude are better with sealevel values of [Hb] than with the high [Hb] values. Whatever the reason, there is some advantage to a lower [Hb] on the Tibetan Plateau; we just have to figure out the mechanisms responsible for it.

In the opening sentences of this article, the question was posed as to how and why essentially all humans never exposed to long‐term hypoxia possess a polycythaemic response to the hypoxia of altitude. The only answer that comes to mind is that the kidney, which produces most of the erythropoietin needed to increase [Hb], cannot distinguish between cellular hypoxia from (a) a sabretooth tiger attack causing blood loss and reduced renal blood flow and (b) ascent to altitude reducing cellular $P_{O_{2}}$. We suggest the kidney has been fooled into thinking that ascent to altitude is actually a sabretooth tiger attack causing blood loss that needs to be restored, and the result is a pointless waste of energy producing more red cells, Hb and work for the heart than necessary. Tibetan kidneys have ‘seen the light’ and are correcting the error, raising the question of whether Andean kidneys will eventually follow suit. Recent work in Andeans indeed suggests a similar process may be underway. A protein‐coding variant in one of the HIF pathway genes, the same gene previously associated with Tibetan adaptation and low [Hb] in Tibetans, is associated with relatively lower haematocrit in Andean highlanders (Lawrence et al., 2024). This variant is distinct from Tibetans’, suggesting convergent evolution through different mechanisms.

We would like to return to the finding that normalization of arterial O_2_ concentration over time at Chacaltaya failed to restore ${\dot{V}}_{O_{2}\max}$. The question is, why not? It was not that long ago that the heart (via cardiac output) was declared ‘the’ limiting factor to ${\dot{V}}_{O_{2}\max}$. Well‐controlled animal studies (Brechue et al., 1993; Brechue et al., 1995; Stainsby et al., 1995) showed that perfused, electrically stimulated muscle peak ${\dot{V}}_{O_{2}}$ depended on blood flow pumped into the muscle. Stray‐Gundersen and colleagues showed in dogs that pericardiectomy allowed stroke volume to rise, enhancing both cardiac output and ${\dot{V}}_{O_{2}\max}$ (Stray‐Gundersen et al., 1986). These studies show clearly that cardiac output/muscle blood flow can affect ${\dot{V}}_{O_{2}\max}$. What these studies did not explore is whether altering any other component of the O_2_ transport system from air to mitochondria might also affect ${\dot{V}}_{O_{2}\max}$. It turns out that ${\dot{V}}_{O_{2}\max}$ can indeed be affected by changes in the other components of O_2_ transport, but other than for Hb, that is a story for another day.

Moreover, if the sole control over ${\dot{V}}_{O_{2}\max}$ were indeed cardiovascular, why did a 56% increase in systemic O_2_ delivery over 9 weeks spent at 5260 m produce a mere 9% increase in ${\dot{V}}_{O_{2}\max}$ (Table 1)? Here is the key point: *Acclimatization may normalize arterial O_2_ concentration, but it cannot normalize arterial* $P_{O_{2}}$. That is simple physics: as barometric pressure falls with altitude, $P_{IO_{2}}$ must fall in proportion, which leads to a domino effect for $P_{O_{2}}$ values at all subsequent steps – and no amount of acclimatization can escape that outcome. Steps in O_2_ transport that depend on diffusion depend in turn on $P_{O_{2}}$ gradients at those steps, which will remain lower than at sea level, no matter the time spent at altitude. The laws of diffusion dictate that O_2_ flux = diffusing capacity × $P_{O_{2}}$ gradient. The diffusive steps will therefore have reduced O_2_ throughput capacity, even if diffusing capacity remains normal. Thus, even if delivery of arterial O_2_ into the muscle vascular bed at altitude is almost normalized by polycythaemia, the penultimate O_2_ transport step of diffusion from blood to muscle mitochondria must be compromised by the lower $P_{O_{2}}$ gradient between red cells and mitochondria, lowering the maximal transport rate of O_2_ to the mitochondria (discussed above). That is why ${\dot{V}}_{O_{2}\max}$ cannot be restored over time at altitude by normalizing arterial O_2_ delivery. The validity of this conclusion is underscored simply by the *immediate* restoration of ${\dot{V}}_{O_{2}\max}$ when $P_{O_{2}}$ values were raised by breathing high O_2_ concentration gas, mentioned above. Thus, the possibility that muscle oxidative phosphorylation potential fell over time, preventing normalized arterial O_2_ delivery from enabling a higher ${\dot{V}}_{O_{2}\max}$, is untenable.

So, what lessons might one learn from these stories? One might be that despite years of acceptance, conventional explanations for physiological observations may be incorrect. Another might be that bigger picture analysis of system (rather than individual organ) function combined with relatively simple observations may reveal what is wrong and point to what is right. And finally, you do not have to be a senior scientist to make significant contributions.

## AUTHOR CONTRIBUTIONS

Both authors have read and approved the final version of this manuscript and agree to be accountable for all aspects of the work in ensuring that questions related to the accuracy or integrity of any part of the work are appropriately investigated and resolved. All persons designated as authors qualify for authorship, and all those who qualify for authorship are listed.

## CONFLICT OF INTEREST

None declared.
